# An update on the neurological short tandem repeat expansion disorders and the emergence of long-read sequencing diagnostics

**DOI:** 10.1186/s40478-021-01201-x

**Published:** 2021-05-25

**Authors:** Sanjog R. Chintalaphani, Sandy S. Pineda, Ira W. Deveson, Kishore R. Kumar

**Affiliations:** 1 School of Medicine, University of New South Wales, Sydney, 2052 Australia; 2 Kinghorn Centre for Clinical Genomics, Garvan Institute of Medical Research, Darlinghurst, NSW 2010 Australia; 3 Garvan-Weizmann Centre for Cellular Genomics, Garvan Institute of Medical Research, Darlinghurst, NSW 2010 Australia; 4 Brain and Mind Centre, University of Sydney, Camperdown, NSW 2050 Australia; 5 Faculty of Medicine, St Vincent’s Clinical School, University of New South Wales, Sydney, NSW 2010 Australia; 6 Molecular Medicine Laboratory and Neurology Department, Central Clinical School, Concord Repatriation General Hospital, University of Sydney, Concord, NSW 2137 Australia

**Keywords:** Tandem, Repeats, Expansion, Neurological, Clinical, Genetics, Disease, Diagnosis, Long-read, Sequencing

## Abstract

**Background:**

Short tandem repeat (STR) expansion disorders are an important cause of human neurological disease. They have an established role in more than 40 different phenotypes including the myotonic dystrophies, Fragile X syndrome, Huntington’s disease, the hereditary cerebellar ataxias, amyotrophic lateral sclerosis and frontotemporal dementia.

**Main body:**

STR expansions are difficult to detect and may explain unsolved diseases, as highlighted by recent findings including: the discovery of a biallelic intronic ‘AAGGG’ repeat in *RFC1* as the cause of cerebellar ataxia, neuropathy, and vestibular areflexia syndrome (CANVAS); and the finding of ‘CGG’ repeat expansions in *NOTCH2NLC* as the cause of neuronal intranuclear inclusion disease and a range of clinical phenotypes. However, established laboratory techniques for diagnosis of repeat expansions (repeat-primed PCR and Southern blot) are cumbersome, low-throughput and poorly suited to parallel analysis of multiple gene regions. While next generation sequencing (NGS) has been increasingly used, established short-read NGS platforms (e.g., Illumina) are unable to genotype large and/or complex repeat expansions. Long-read sequencing platforms recently developed by Oxford Nanopore Technology and Pacific Biosciences promise to overcome these limitations to deliver enhanced diagnosis of repeat expansion disorders in a rapid and cost-effective fashion.

**Conclusion:**

We anticipate that long-read sequencing will rapidly transform the detection of short tandem repeat expansion disorders for both clinical diagnosis and gene discovery.

## Introduction

A large proportion of the human genome is comprised of repetitive DNA sequences known as microsatellites or short tandem repeats (STRs). STRs are small sections of DNA, usually 2–6 nucleotides in length, that are repeated consecutively at a given locus. STRs make up at least 6.77% of the human genome and are highly polymorphic [143]. STR lengths are prone to alteration during DNA replication, due to slippage events on misaligned strands, errors in DNA repair during synthesis and formation of secondary hairpin structures [43]. As a result, STR lengths are relatively unstable, with their frequent mutation providing a source of genetic variation in human populations. STRs have a mutation rate orders of magnitude higher than single nucleotide polymorphisms (SNPs) in non-repetitive contexts [58]. Larger repeats, in general, are more unstable and have an increased propensity to expand during DNA replication.

Large STR expansions may become pathogenic, underpinning various forms of primary neurological disease. There are currently 47 known STR genes that can cause disease when expanded; 37 of these exhibit primary neurological presentations (see Table 1) while 10 present with developmental abnormalities (see Table 2). With increased interest and improving molecular techniques for detecting repeat expansions, the list of known repeat expansion disorders is growing rapidly, with new genes such as *RFC1*, *GIPC1*, *LRP12*, *NOTCH2NLC* and *VWA1* recently implicated. Furthermore, STR expansions have been linked to complex polygenic diseases such as heart disease, bipolar disorder, major depressive disorder and schizophrenia [59]. Some theories also suggest STR variability may account for normal brain and behavioural traits such as anxiety, cognitive function, emotional memory and altruism [41]. Similarly, somatic instability at STR regions is a hallmark of many cancers such as Lynch syndrome-related cancers, gastric cancers, colorectal cancers and endometrial cancers [174]. In this review, we provide an overview of the primary neurological repeat expansion diseases, discuss limitations in current diagnostic methods and developments in long-read sequencing technologies that promise to improve the discovery and diagnosis of STR expansions.

**Table 1:** Summary of known neurological diseases caused by short tandem repeat expansions

Abbreviated phenotype (MIM number)	Gene	Mode of inheritance	Repeat Motif	Location on Gene	Pathogenic repeat number^a^	Chromosome	Coordinates (hg38)		Clinical phenotype	References
C9-FTD C9-ALS (#10550)	*C9orf72*	AD	GGGGCC	5’ Region	24–4000	chr9	27573485	27573546	Frontotemporal dementia, amyotrophic lateral sclerosis	[32, 47, 65]
CANVAS (#614575)	*RFC1*	AR	(AAGGG)_400–2000_ (ACAGG)_exp_ AAAAG (normal)	Intron 2	400–2000	chr4	39348425	39348483	Cerebellar ataxia, neuropathy, and vestibular areflexia syndrome	[11, 28, 138]
DM1 (#160900)	*DMPK*	AD	CTG (Interruptions: CCG)	3’ Region	50–10,000	chr19	45770205	45770266	Myotonic dystrophy 1	[60, 176]
DM2 (#602668)	*CNBP* (*ZNF9*)	AD	CCTG	Intron 1	50–11,000	chr3	129172577	129172656	Myotonic dystrophy 2	[176]
DRPLA (#125370)	*ATN1*	AD	CAG	Exon 5	49–93	chr12	6936717	6936775	Dentatorubral-pallidoluysian atrophy	[78]
EIEE1/XLID (#308350) (#300419) (#300215)	*ARX*	XL	GCC	Exon 2	17–27	chrX	25013654	25013697	Clinical spectrum of disorders including developmental and epileptic encephalopathy 1, hydranencephaly with abnormal genitalia, X-linked lissencephaly 2 and X-linked mental retardation 29	[73, 150]
FAME1 (#601068)	*SAMD12*	AD	TTTCA within TTTTA repeat region	Intron 4	105–3680	chr8	118366813	118366918	Familial adult myoclonic epilepsy 1	[22, 68]
FAME2 (#607876)	*STARD7*	AD	ATTTC within ATTTT repeat region	Intron 1	150–460	chr2	96197067	96197124	Familial adult myoclonic epilepsy 2	[27]
FAME3 (#613608)	*MARCHF6*	AD	TTTCA within TTTTA repeat region	Intron 1	700–1035	chr5	10356339	10356411	Familial adult myoclonic epilepsy 3	[40]
FAME6 (#618074)	*TNRC6A*	AD	TTTCA within TTTTA repeat region	Intron 1	? (only 1 family)	chr16	24613439	24613532	Familial adult myoclonic epilepsy 6	[68]
FAME7 (#618075)	*RAPGEF2*	AD	TTTCA within TTTTA repeat region	Intron 14	? (only 1 family)	chr4	159342527	159342618	Familial adult myoclonic epilepsy 7	[68]
FRAXE (#309548)	*FMR2* (*AFF2*)	XLR	CCG	5’ Region	> 200	chrX	148500605	148500753	Mental retardation, X-linked, FRAXE type	[53]
FRDA (#229300)	*FXN*	AR	GAA	Intron 1	66–1300	chr9	69037275	69037314	Friedreich ataxia	[5, 19, 162]
FXS (#300624) FXTAS (#300623)	*FMR1*	XL	CGG	5’ Region	200–3000 55–200	chrX	147911979	147912111	Fragile X syndrome Fragile X tremor/ataxia syndrome, premature ovarian failure 1	[162] [56]
HD (#143100)	*HTT*	AD	CAG (Interruptions: CAA)	Exon 1	36–250	chr4	3074876	3074941	Huntington disease	[96, 101]
HDL1 (#603218)	*PRNP*	AD	24-base octapeptide PHGGGWGQ	Exon 2	8–14	chr20	4699379	4699380	Huntington disease-like 1	[108]
HDL2 (#606438)	*JPH3*	AD	CTG	Exon 2A	40–59	chr16	87604283	87604329	Huntington disease-like 2	[62]
HMN	*VWA1*	AR	GGCGCGGAGC	Exon 1	3	chr1	1435799	1435820	Hereditary axonal motor neuropathy	[121]
NIID (#603472)	*NOTCH2NLC*	AD	CGG	5' Region	66–517	chr1	149390803	149390842	Neuronal intranuclear inclusion disease	[55, 118, 146]
OPDM1 (#164310)	*LRP12*	AD	CGG	5' Region	90–130	chr8	104588965	104588999	Oculopharyngodistal myopathy	[69]
OPDM2 (#618940)	*GIPC1*	AD	CGG	5’ Region	70–164	chr19	14496029	14496104	Oculopharyngodistal myopathy	[172]
OPMD (#164300)	*PABPN1*	AD	GCG	Exon 1	7–18	chr14	23321472	23321511	Oculopharyngeal muscular dystrophy	[15, 129]
OPML1 (#618637)	*NUTM2B-AS1*	AD	CGG	5' Region	16–160	chr10	79826364	79826403	Oculopharyngeal myopathy with leukoencephalopathy 1	[69]
SBMA (#313200)	*AR*	XLR	CAG	Exon 1	38–68	chrX	67545317	67545419	Spinal and bulbar muscular atrophy of Kennedy (Kennedy's disease)	[44, 82, 147]
SCA1 (#164400)	*ATXN1*	AD	CAG (Interruptions: CAT)	Exon 8	39–91	chr6	16327636	16327723	Spinocerebellar ataxia 1	[120, 141]
SCA2 (#183090)	*ATXN2*	AD	CAG (Interruptions: CAA, CGG, CGC)	Exon 1	33–200 (29–32 increased ALS risk)	chr12	111598950	111599019	Spinocerebellar ataxia 2	[18, 133, 141, 148]
SCA3 (#109150)	*ATXN3*	AD	CAG	Exon 10	53–87	chr14	92071011	92071052	Spinocerebellar ataxia 3	[74]
SCA6 (183086)	*CACNA1A*	AD	CAG	Exon 47	19–33	chr19	13207858	13207897	Spinocerebellar ataxia 6	[141, 181]
SCA7 (#164500)	*ATXN7*	AD	CAG	Exon 1	34–460	chr3	63912685	63912716	Spinocerebellar ataxia 7	[18, 30]
SCA8 (#608768)	*ATXN8*	AD	CAG/TAG	3’ UTR	74–1300	chr13	70139383	70139428	Spinocerebellar ataxia 8	[79, 141, 155]
SCA10 (#603516)	*ATXN10*	AD	ATTCT (Interruptions: ATCCT)	Intron 9	280–4500	chr22	45795355	45795424	Spinocerebellar ataxia 10	[88, 100, 141]
SCA12 (#604326)	*PPP2R2B*	AD	CAG	5’ Region	51–78	chr5	146878729	146878758	Spinocerebellar ataxia 12	[63, 94, 141]
SCA17 (#607136)	*TBP*	AD	CAG (Interruptions: CAT, CAA)	Exon 3	43–66	chr6	170561907	170562017	Spinocerebellar ataxia 17, Huntington disease-like 4	[97, 115, 141]
SCA31 (#117210)	*BEAN1*	AD	TGGAA within TAAAA and TAGAA repeat region	Intron/ Intergenic region	500–760 (> 110 TGGAA repeats)	chr16	66495475	66495509	Spinocerebellar ataxia 31	[134]
SCA36 (#614153)	*NOP56*	AD	GGCCTG	Intron 1	650–2500	chr20	2652733	2652775	Spinocerebellar ataxia 36	[77]
SCA37 (#615945)	*DAB1*	AD	ATTTC within (ATTTT)_7–400_ repeat region	5’ Region	31–75	chr1	57367044	57367125	Spinocerebellar ataxia 37	[139]
ULD (#254800)	*CSTB*	AR	CCCCGCCCCGCG	Upstream 5’ UTR	30–125	chr21	43776444	43776479	Progressive myoclonic epilepsy 1A (Unverricht and Lundborg disease)	[87, 91]

ALS, amyotrophic lateral sclerosis; AS, antisense RNA; CANVAS, cerebellar ataxia neuropathy and vestibular areflexia syndrome; DM1; myotonic dystrophy 1; DM2; myotonic dystrophy 2; DRPLA, dentatorubral-pallidoluysian atrophy; EIEE1, early infantile epileptic encephalopathy 1; FAME, familial adult myoclonic epilepsy; FRAXE, fragile-XE syndrome; FRDA, Friedreich’s ataxia; FTD, frontotemporal dementia; FXS, fragile-X syndrome; FXTAS, fragile-x tremor/ataxia syndrome; HMN, hereditary motor neuropathy; HD, Huntington’s disease; HDL2, Huntington disease-like 2; HDL1, Huntington disease-like 1; LMN, lower motor neuron; NIID, neuronal intranuclear inclusion disease; OPDM, oculopharyngodistal myopathy; OPMD, oculopharyngeal muscular dystrophy; OPML, oculopharyngeal myopathy with leukoencephalopathy; SBMA, spinal and bulbar muscular atrophy; SCA, spinocerebellar ataxia; ULD, Unverricht-Lundborg disease; UMN, upper motor neuron; XLID, x-linked intellectual disability;

^a^These ranges vary between studies and often the upper limit is unknown. It is important to note that these are only potentially pathogenic. There is a small (< 1%) subsection of the healthy control population who have expanded alleles with no clinical manifestations. Similarly, there are alleles lower than the given range who may have intermediate alleles and premutation syndromes

**Table 2:** Summary of known congenital and developmental disorders caused by short tandem repeat expansions. Adapted from Khristich and Mirkin [76]

Phenotype (OMIM #)	Gene	Motif	Pathogenic repeat number	Location	(hg38)			References
BPES (#110100)	*FOXL2*	GCG	22–24	Exon	chr3	138946022	138946062	[116]
CCHS (#209880)	*PHOX2B*	GCG	24–33	Exon	chr4	41745976	41746022	[7]
DBQD2 (#615777)	*XYLT1*	GGC	100–800	5’ Region	chr16	17470869	17470967	[86]
FECD3 (#613267)	*TCF4*	TGC	> 50	Intron	chr18^a^	55222184^a^	55635956^a^	[167]
GDPAG (#618412)	*GLS*	GCA	> 300	5’ Region	chr2	190880873	190880920	[159]
HFG (#140000)	*HOXA13*	GCG	24–26	Exon	chr7	27199827	27199967	[50]
HPE5 (#609637)	*ZIC2*	GCG	25	Exon	chr13	99985449	99985494	[17]
HSAN8 (#616488)	*PRDM12*	GCG	18–19	Exon	chr9	130681606	130681641	[23]
SPD1 (#186000)	*HOXD13*	GCG	22–29	Exon	chr2	176093058	176093099	[2]
XLMR (#300123)	*SOX3*	GCG	15–26	Exon	chr3	181712415	181712456	[89]

BPES, blepharophimosis, epicanthus inversus, and ptosis; CCHS, congenital central hypoventilation syndrome; DBQD2, Desbuquois dysplasia 2; FECD3, Fuchs endothelial corneal dystrophy 3; GDPAG, global developmental delay, progressive ataxia, and elevated glutamine; HFG, hand-foot-genital syndrome; HPE5, holoprosencephaly 5; SPD1, synpolydactyly 1; XLMR, x-linked mental retardation

^a^Location of entire gene listed

## General characteristics of repeat expansion disorders

### Molecular mechanisms

Repeat expansion diseases have a wide range of pathogenic mechanisms, which depend on the location of the expanded STR within a gene loci, and the nature and function of the gene. It is often hard to determine the specific mechanism as multiple may occur simultaneously and all may contribute to the disease form. The mechanisms may be broadly categorised as loss-of-function (LOF) or toxic gain-of-function (GOF).

LOF mechanisms include hypermethylation and gene silencing [43, 132], defective transcription, and increased messenger RNA (mRNA) degradation [154]; all effects that can be elicited by an STR expansion within a gene locus. DNA methylation is an epigenetic process that contributes to genome stability and maintenance, and regulation of gene expression during development, with aberrant methylation profiles often implicated in disease [2]. Large expanded STRs may induce local hypermethylation, thereby silencing gene expression. One such classic example is an expanded STR in the promoter region of *FMR1*, seen in Fragile X syndrome (FXS). The expansion causes hypermethylation of the *FMR1* promoter region leading to silencing of transcription and LOF in the *FMR1* gene. Therefore, the methylation state of relevant genes, in addition to STR length, may be informative for diagnosis of repeat expansion diseases.

Toxic GOF mechanisms include RNA toxicity, aberrant alternative splicing, repeat-associated non-AUG (RAN) translation, increased promoter activity, coding tract expansions and polyglutamine aggregation [85, 154, 180]. Repeat expansions in coding and non-coding regions may disrupt RNA function in many ways, with multiple coexisting mechanisms potentially contributing to pathogenicity. For example, post-mortem examination of brain tissue in patients with an expanded ‘GGGGCC’ repeat in the 5’ region of *C9orf72* ALS/FTD, revealed multiple potential pathogenic RNA species: RNA that had been stalled at repeat locations, RAN proteins, antisense transcription of repeat regions and alternative splicing of intron 1 containing the repeat [48]. These species are considered “toxic” as they accumulate as RNA foci within the neurons, astrocytes, microglia and oligodendrocytes and form complexes with RNA-binding proteins to dysregulate translation and modify transcription [48, 49].

The other common toxic GOF mechanism is expansion of homopolymer amino acid tracts resulting in misfolding and proteinopathy. In neurological repeat expansion diseases, exonic ‘CAG’ repeat expansions code for the amino acid glutamine; when expanded, they create polyglutamine tract expansions which can reach hundreds of amino acids long. This is thought to alter and expand the transcribed protein creating insoluble protein aggregates within neuronal cells (primarily in the cerebellum), leading to perturbations of intracellular homeostasis and cell death [81]. This mechanism is commonly seen in the hereditary spinocerebellar ataxias. In congenital and developmental repeat expansion diseases, exonic ‘GCG’ coding tracts expand to create polyalanine tract expansions (Table 2). However, they are quite different to polyglutamine tract expansions seen in neurological repeat expansion disorders; they are smaller and generally meiotically stable when transmitted between generations, thus they do not exhibit the same large pathogenic range seen in neurological repeat expansion disorders. For example, a normal allele in *HOXA13* contains 15–18 alanine residues while a pathogenic allele only contains between 7 and 15 extra residues [50]. Thus, the mechanism of mutation in polyalanine disorders is thought to be different and hypothesised to be due to unequal crossing between mispaired alleles and duplication during replication rather than dynamic trinucleotide expansions [164]. This would explain the relative stability of transmission and small pathogenic ranges. Furthermore, these polyalanine tract repeat expansion disorders are more commonly caused by other mutations such as missense and frameshift mutations. Interestingly, several studies show that an expansion of polyalanine tracts results in low levels of the protein found in the nucleus thereby exhibiting LOF, rather than increased protein levels and proteinopathy seen in polyglutamine tract expansions [23, 64].

### Repeat length and disease severity

**Fig. 1:**
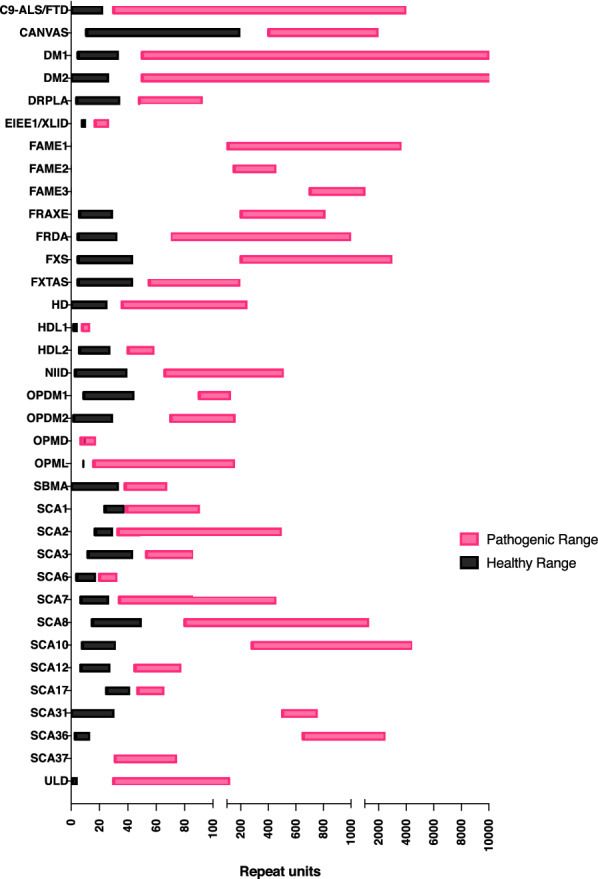
Healthy and pathogenic ranges in neurological short tandem repeat expansion disorders. Box plot indicates the range of observed sizes for the pathogenic STR in known neurological STR expansion disorders (see Table 1). For each disorder, the range of STR sizes observed among unaffected individuals is shown in black, and the sizes observed in affected individuals is shown in pink

The size of STR expansions has been shown to quantitively affect disease severity, with larger expansions often associated with earlier onset of disease and more severe symptoms. For example, the repeat size in myotonic dystrophy type 1 (DM1) has a very broad pathogenic range (Fig. 1). Typically, 50–150 repeats cause a late-onset (20–70 years) mild phenotype with cataracts and myotonia, 100–1000 repeats cause onset in adolescence/early adulthood (10–30 years) with a classical phenotype of weakness, myotonia, cataracts, balding and arrhythmias, while even larger expansions cause early-onset (birth to 10 years) disease with infantile hypotonia, respiratory involvement and intellectual disability [13, 176].

Slightly expanded STR regions, known as premutation alleles, may be associated with mild or variable phenotypes. For example, in Huntington’s disease (HD), there is full penetrance in all individuals with greater than 39 repeats of ‘CAG’ within exon 1 of the *HTT* gene, and partial penetrance in individuals with 36–39 repeats [101]. Approximately 50–70% of the variability in age of onset in Huntington’s disease is directly correlated to repeat length variability [54, 170]. Another classical example is FXS. In 1991, it was found that a ‘CGG’ repeat in the 5’ promoter region of the *FMR*1 gene normally contains an unmethylated STR of up to 45 ‘CGG’ repeats [55]. In individuals with expansions greater than 200 repeats, the *FMR1* promoter region undergoes hypermethylation and transcriptional silencing of Fragile X mental retardation protein (FMRP) [109]. Loss of the FMRP protein, which is vital for synaptic plasticity in the CNS, leads to FXS [10]. However, the premutation allele (55–200 repeats) is known to cause late-onset Fragile X-associated tremor/ataxia syndrome (FXTAS) in men [90]. While in women, a 55–200 repeat-allele may present with a primary ovarian insufficiency due to absent menarche or premature follicular depletion [109]. This premutation allele does not exhibit hypermethylation, and in fact increases promoter region activity and transcription, resulting in production of toxic RNA species [59]. Thus, two allele sizes in the same STR region may exhibit opposing molecular mechanisms corresponding with distinct clinical phenotypes. This highlights the importance of accurate repeat sizing for these genes.

It is important to note that the exact point at which STR pathogenicity occurs is still the subject of ongoing investigation and debate. For example, there is some uncertainty over the pathogenic cut-off for SCA8 and SCA17, since expanded alleles have been detected in a healthy control population [142, 178]. Moreover, the pathogenic link between the STR expansion in *ATXN8* and SCA8 has been questioned [136, 149, 169]. Rates of expanded repeats in healthy populations exist in other STR regions, such as *C9orf72* and *FMR1*, where 0.1–0.4% of the healthy population have a repeat expansion [69]. Hence, in these cases it is difficult to determine the significance of an expanded or slightly expanded allele. Furthermore, due to intrinsic limitations in current clinical diagnostic methods, the upper range of STR expansions is often difficult to accurately define, with large expansions exceeding the capabilities of established molecular diagnostic techniques (see below). For example, the sizing of SCA31 repeats has been imprecise or absent, with no accurate literature defining the upper end of pathological repeat sizes [67]. Generally, genetic reports for *C9orf72* indicate three size ranges: normal, intermediate and pathogenic [16]. The pathogenic range is generally reported as “ > 30” repeats [16].

### Clinical anticipation

As mentioned earlier, STRs have an intrinsic tendency to expand during replication. This means that, while most repeat expansion diseases are inherited, there may be sporadic cases with no previous family history. STR instability also explains a phenomenon known as clinical anticipation. Anticipation is the seemingly increasing severity of disease and/or symptoms appearing at an earlier age as generations continue. Because of this phenomenon, the premutation allele in FXS is commonly seen in maternal carriers and maternal grandfathers of affected individuals. Over generations, the unstable premutation allele favours continual expansion and may sporadically present as full FXS in male children. Anticipation is also commonly seen in HD, with larger repeats being more unstable [130]. Intermediate alleles of 34–35 ‘CAG’ repeats in *HTT* have a high risk of expanding and causing new mutations [140]. Interestingly, anticipation in HD is much more commonly seen in paternal transmission, with larger expansion juvenile-onset HD often inherited from the father; although, there are some cases of maternal transmission [113, 127]. This is thought to be due to large STR instability and variation in spermatogenesis seen in fathers [166]. This paternal transmission pattern of anticipation is also seen in SCA1, SCA2, SCA7 and DRPLA [6, 51, 66, 99], while in SCA8 there is a pattern of maternal transmission thought to be due to en masse STR contractions in paternal sperm [110]. *ATN1* (DRPLA) and *ATXN7* (SCA7) are especially unstable [125]; anticipation in SCA7 may be so severe that young children develop symptoms before an affected parent or grandparent.

The phenomenon of genetic anticipation may not be true for all repeat expansion diseases, for example, clinical anticipation is not seen in families with OPMD or FRDA [52, 71], and while studies show evidence of clinical anticipation in *C9orf72* expanded alleles [160], carrier alleles may variably contract or expand over generations [42]. Furthermore, the repeat length has been found to differ within the same patient, indicating cells in brain tissue and cells in blood have different repeat sizes (similar patterns of somatic mutation are seen in other repeat expansion disorders such as HD and DM1) [123]. Thus, further accurate genotyping of *C9orf72* affected families is required to better understand the correlation between repeat size and phenotype.

### Common clinical features

Repeat expansion diseases tend to cluster around shared phenotypes. It would be difficult to find a repeat expansion disorder that did not exhibit of one or more of the following phenotypes: cerebellar ataxia, chorea or HD phenocopies, tremor, cognitive impairment, muscular dystrophies, myoclonic seizures, amyotrophic lateral sclerosis and peripheral neuropathies.

#### Hereditary cerebellar ataxias

Patients with hereditary cerebellar ataxia exhibit abnormal eye movements, dysarthria, limb and gait ataxia. These may be due to a plethora of different STR expansions including the spinocerebellar ataxias (SCA), dentatorubral-pallidoluysian atrophy (DRPLA), Friedreich’s Ataxia (FRDA) and the cerebellar ataxia, neuropathy, and vestibular areflexia syndrome (CANVAS, see section below) [12], and may also be due to point mutations, duplications, and deletions [71].

The most common STR expansions in patients with hereditary cerebellar ataxia is an expanded ‘CAG’ repeat within polyglutamine tracts found in SCA1, SCA2, SCA3, SCA6, SCA7, SCA12 and SCA17 [131]. For these disorders, there are efficient cost-effective repeat-primed polymerase chain reaction (RP-PCR) methods for diagnostic testing, however a majority of patients referred for these panels return with negative test results [72]. Testing other STR regions is not as straight forward, and requires time-consuming methods of individual gene sequencing [8]. In a German cohort of 440 of people who returned negative for SCA1, 2, 3, 6 and 7, there were five patients with expanded SCA8 repeats, one patient with an FXTAS expanded allele and four with possible FXTAS alleles, and one *C9orf72* expansion [8]. This study shows that, while they are uncommon, other STR expansions may cause undiagnosed late-onset progressive ataxia. Recently, SCA37 was linked to a novel expansion of ‘ATTTC’ within a ‘ATTTT’ polymorphism in *DAB1* [139]. The repeat length and conformation of the repeat expansion could only be accurately assessed with long-read sequencing [139]. It has a similar phenotype to other spinocerebellar ataxias, suggesting there are more novel expansions which may explain cases of undiagnosed ataxia.

#### Myoclonus epilepsies

Unverricht-Lundborg disease (ULD) is one of the most common single causes of progressive myoclonus epilepsy worldwide; it is characterised by childhood-onset stimulus-sensitive myoclonus epilepsy, ataxia and cognitive and behavioural abnormalities [91]. Other repeat expansion diseases may also present with myoclonus epilepsies, usually with large repeat sizes and severe phenotypes; these include SCA7, SCA10  and DRPLA [92, 103, 161, 177]. Furthermore, a group of familial adult myoclonus epilepsies (FAME1, 2, 3 and 6) have recently been linked to STR expansions, discussed further below.

#### Huntington’s disease and Huntington’s disease phenocopies

HD is caused by a ‘CAG’ repeat in the *HTT* gene and is characterised by chorea with psychiatric symptoms and cognitive decline, with mean age of symptom onset between 35 to 44 years old [20]. The most common HD phenocopies or HD-like syndromes are seen in STR expansions within *C9orf72* [111] (discussed below), however, others include *PRNP* (Huntington disease-like 1, HDL1), *JPH3* (HDL2), *TBP* (SCA17 or HDL4), *ATXN8* (SCA8), *FXN* (Friedreich’s ataxia) and *ATN1* (DRPLA), in addition to sequencing variants/deletions in *VPS13A*, *TITF1*, *ADCY5*, *RNF216* and *FRRS1L* [135]. HDL2 shares molecular characteristics with HD: they are both due to polyglutamine tract expansion caused by a ‘CAG’ repeat in exon 1 of their respective genes, and there is evidence to suggest that similar CREB-binding protein (CBP) sequestration in nuclear bodies drives both pathological processes [62, 168]. Given numerous examples of HD phenocopies and the overlap between several repeat expansion diseases, one may suspect that further phenocopies of HD might have an undiscovered genetic basis in STR regions.

#### *C9orf72*-related disorders

Since its discovery in 2011, the ‘GGGGCC’ hexanucleotide repeat in *C9orf72* has been studied extensively. It is the most common cause of familial frontotemporal dementia (FTD) and familial amyotrophic lateral sclerosis (ALS) [32]. Interestingly, the *C9orf72* repeat expansion has also been linked to a range of clinical phenotypes including typical Parkinson’s disease, atypical parkinsonian syndromes, schizophrenia and bipolar disorder [14, 49]. In a recent retrospective study, movement disorders were the second most common initial presentation of *C9orf72*-related diseases, following cognitive signs in FTD [37]. These patients frequently present with one or several of the following: parkinsonism, myoclonus, dystonia, chorea and ataxia [37]. The phenotypic heterogeneity is difficult to explain, consistent with the concept that the mechanisms of disease caused by STR expansions are poorly understood [59].

### Interruptions

Some STR expansions contain internal sequence interruptions that may directly affect the phenotype or lead to overestimation of repeat sizes. These interruptions have long been found in Fragile X, Huntington’s disease, hereditary cerebellar ataxias and myotonic dystrophies, however their origins and effect are poorly understood. There has been more research in this area due to new methods of long-read sequencing, combined with specific RP-PCR and Southern blot primers to establish a stronger consensus on repeat motifs [156]. This has allowed new discoveries in the role of interruptions. For example, three groups have shown that a loss of a ‘CAA’ interruption within expanded ‘CAG’ tracts in *HTT* leads to earlier onset Huntington’s disease [170]. It is estimated that this variant is associated with 9.5 years earlier onset in Huntington’s disease [39], particularly in those with reduced penetrance alleles of 36–39 ‘CAG’ repeats. The ‘CAA’ interruption is also a genetic modifier of other polyglutamine repeat expansions, such as SCA2 and SCA17 [25, 45]. These ‘CAA’ interruptions fall within ‘CAG’ coding tracts and therefore still translate to glutamine, however the interrupted alleles preferentially form shorter branching hairpin structures which reduce strand slippage and increase stability of the repeat [145, 173]. Thus, it is proposed that the pathogenic mechanism of this interruption may be due to increased instability during somatic expansion of the repeat, and longer polyglutamine tracts leading to increased toxic GOF [170]. Interestingly, in SCA2, ‘CAA’, ‘CGG’ and ‘CGC’ interruptions are linked to autosomal dominant levodopa-responsive Parkinson’s disease, demonstrating interruptions may modify phenotype as well as age of onset [122].

Similarly, a DM1 family was found to have ‘CCG’ interruptions within the ‘CTG’ STR expansion in *DMPK* resulting in atypical traits such as severe axial and proximal weakness and late onset of symptoms [9].

Pentanucleotide STR regions are very unstable and dynamic in nature, often containing large amounts of heterogeneity in controls as well as patients. For example, pathogenic ‘ATTCT’ repeats in *ATXN10* (SCA10) likely exist within a dynamic structure of pentanucleotide, hexanucleotide and heptanucleotide motifs [102]. Interruptions with the specific ‘ATCCT’ motif is strongly associated with epilepsy [88, 103], while pure ‘ATTCT’ tracts are associated with parkinsonism [137]. The mechanism of disease caused by these interruptions is difficult to discern; further genotyping of these regions is first required. This complex motif structure is commonly seen in several newly discovered pentanucleotide repeat expansions such as *RFC1* or *SAMD12*, which show that pathogenic sequences are often extremely dynamic in nature [3, 107, 138].

## Recent discoveries for neurological repeat expansion disorders

Most of the repeat expansion disorders listed in Table 1 have been discussed extensively in literature, however, in the last three years, 12 novel neurological repeat expansion disorders have been classified – these include SCA37, CANVAS, neuronal intranuclear inclusion disease (NIID), OPML, OPDM, OPDM2, FAME1, FAME2, FAME3, FAME6, FAME7 and recessive hereditary motor neuropathy (HMN) (Table 1).

In 2019, a heterozygous ‘CGG’ expansion in the *Notch homolog 2N-terminal-like C* (*NOTCH2NLC*) gene was found to be the cause of NIID by numerous independent groups [34, 69, 146]. Of note, the expansion was detected or confirmed using long-read sequencing. Some patients have been identified to have ‘AGG’ interruptions, with evidence in a small East–Asian cohort showing interruptions may be linked to earlier age of onset [24]. NIID is a neurodegenerative condition characterized by eosinophilic intranuclear inclusions in neuronal and glial cells, which have characteristic findings on brain MRI, including high diffusion-weighted imaging signals along the corticomedullary junction [4, 95, 152]. The *NOTCH2NLC* expansion has also been found in a rapidly growing number of phenotypes, including leukoencephalopathy, essential tremor, Parkinson’s disease, multiple system atrophy (MSA) and amyotrophic lateral sclerosis [38, 69, 95, 117, 119, 175]. Further long-read sequencing studies have found noncoding CGG repeat expansions in *LOC642361/NUTM2B-AS1*, *LRP12* and *GIPC1* [69, 172]. These STR expansions correspond to similar phenotypes: oculopharyngeal myopathy with leukoencephalopathy (OPML), and oculopharyngodistal myopathy 1 and 2 (OPDM1 and OPDM2), emphasising the need for screening multiple genetic causes in patients presenting with these clinical features. For example, a recent study screened a cohort of 211 patients clinically diagnosed with OPDM and found seven patients with ‘CGG’ expansions in *NOTCH2NLC* [118]. Similarly, in a cohort of 189 patients clinically diagnosed with MSA, five were found to have ‘GCC’ repeats in *NOTCH2NLC* [38].

In 2019, an intronic biallelic ‘AAGGG’ repeat in the *RFC1* gene was linked to patients presenting with cerebellar ataxia, neuropathy and vestibular areflexia syndrome (CANVAS) [28, 126]. CANVAS is characterised by a collection of clinical features which often present later in life [21]. Previously determined idiopathic [171], the newly discovered repeat expansion was found in 22% of all patients (n = 150) with undiagnosed late-onset ataxia. This percentage increased to 63% if they also had sensory neuronopathy and up to 92% of patients with full CANVAS syndrome features [28], however these numbers seem to be an overestimation in non-European populations [3]. *RFC1* expansions can also mimic other disorders such as Sjogren’s syndrome, hereditary sensory neuropathy with cough or paraneoplastic syndrome [29, 83]. Interestingly, in this case, the pathogenic repeat ‘AAGGG’ is  a conformational variation on the normal ‘AAAAG’ motif, suggesting a disease mechanism associated with the expansion of variant motifs. Many studies have shown the dynamic nature of the repeats within *RFC1*. A study of 608 healthy controls used flanking and RP-PCR, Southern blot analysis and Sanger sequencing to demonstrate an allelic distribution of 75.5% for the ‘(AAAAG)_11_’ allele, 13.0% for the ‘(AAAAG)_exp_’ allele, 7.9% for the ‘(AAAGG)_exp_’ allele and 0.7% for the ‘(AAGGG)_exp_’ allele [28]. The average size of normally expanded alleles ‘AAAAG’ and ‘AAAGG’ was 15–200 repeats and 40–1000 repeats respectively. Another study reports two other heterozygous conformations, ‘AAGAG’ and ‘AGAGG’, which have an average size of 160 repeats and a frequency of approximately 2% in healthy populations and 7% in CANVAS cases [3].

Recently, more novel pathogenic *RFC1* conformations have been implicated with CANVAS. ‘ACAGG’ was found to have expanded in two Asia–Pacific families [138] who demonstrated additional clinical features, namely fasciculations and elevated serum kinase. Another study showed a ‘(AAAGG)_10–25_(AAGGG)_exp_’ allele was the predominant pathogenic allele found in Māori populations, with no apparent phenotypic differences when compared to the European populations [11]. Accurately genotyping the conformation of the expanded allele in *RFC1* is vital for diagnosing CANVAS and discovering novel pathogenic conformations. Long-read sequencing has been used to read entire lengths of repeat regions and overcomes traditional problems of mapping novel conformations with short-reads or creating repeat-primed probes with RP-PCR and Southern blot. This is also seen in SCA37 and the five FAME subtypes, whereby a variant conformation is expanded within the patient cohort [68, 139].

In 2019, five subtypes of familial adult myoclonus-epilepsies (FAME) were linked to ‘TTTCA’ intronic repeats in their respective genes [68]. Using PacBio long-read sequencing, the 2.2–18.4 kb expanded alleles in *SAMD12* (FAME1) could be accurately and efficiently sized [68, 107] and were found to have expanded ‘TTTCA’ segments rather than the ‘TTTTA’ motif found in control patients. FAME6 and FAME7 only have genotype–phenotype linkage in one family each, thus evidence regarding these two diseases is still limited [68].

It is possible a shared motif/repeat location may cause similar clinical syndromes. The ‘TTTCA’ intronic repeats in *SAMD12*, *MARCHF6*, *TNRC6A* and *RAPGEF2* are all responsible for FAME [68]. Similarly, the ‘CGG’ non-coding repeat in NIID, OPML and OPDM also have overlapping phenotypes with some common typical MRI findings.

Very recently, a 10 base pair expansion in the gene *VWA1* was identified as a cause of recessive distal hereditary motor neuropathy (HMN), further underscoring that repeat expansions can be linked with neuropathy phenotypes and highlighting the rapid rate of new STR expansions [121].

**Fig. 2:**
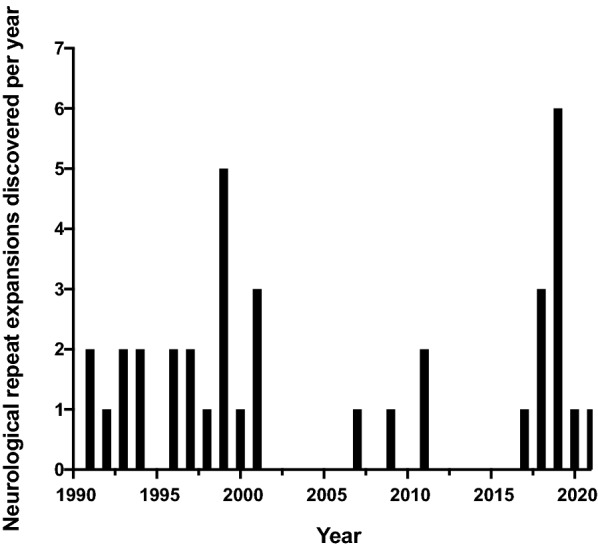
Rate of discovery of neurological short tandem repeat expansions. Bar plot indicates the number of new pathogenic STR expansion discoveries published each year during the period 1990–2021 (see Table 1 for references to original publications for each gene)

Current clinical testing approaches for repeat expansion diseases are time-consuming to develop, and often cannot accurately assess larger STR regions with high ‘GC’ content. We must establish a new robust clinical pipeline for STR genotyping, that can be developed at a rapid pace, to match the rate of discovery of novel repeat expansion diseases as seen in Fig. 2.

## Molecular diagnostics

**Fig. 3:**
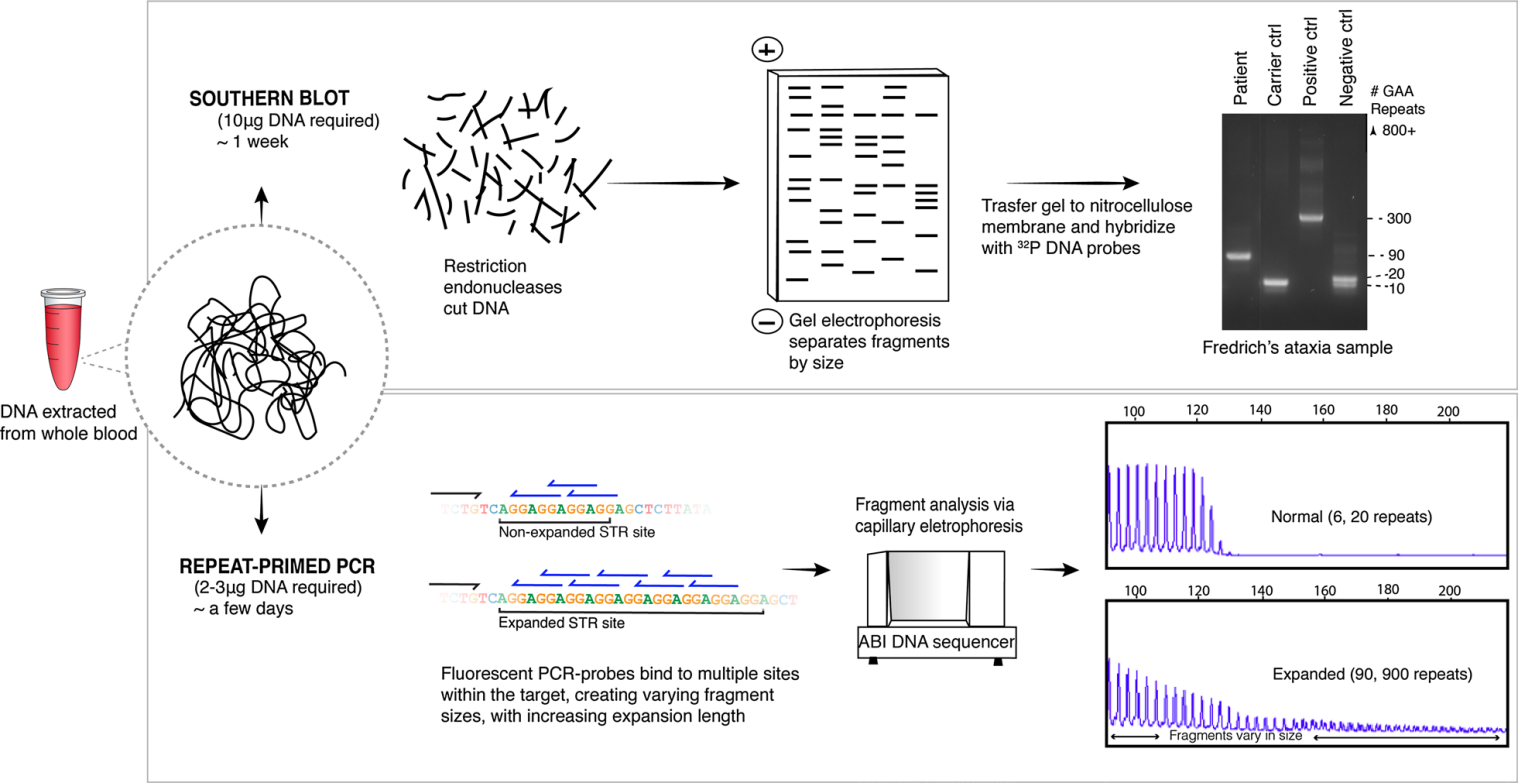
Current molecular diagnostic methods. Flow chart shows an example of two current diagnostic methods for diagnosing STR expansions: Southern blot and repeat-primed PCR. The sample analysis shown in both diagnostic methods was taken from a patient with Friedrich’s ataxia with a heterozygous ‘GAA’ expansion in the *FXN* gene (approximately 90 and 900 repeats). The RP-PCR graph shows the characteristic tailing/stuttering pattern of expanded alleles caused by the repeat-primed probes binding to more sites within the STR expansion. For sizing, Southern blot is performed. The larger 900-repeat ‘GAA’ allele cannot be seen using the Southern blot sizing ladder shown above

The established approach for molecular diagnosis of repeat expansion diseases involves genotyping STRs by repeat-primed precise PCR (RP-PCR) and/or Southern blot assays for sizing larger expansions (Fig. 3). The clinician must decide which STRs warrant testing, which can be difficult due to phenotypic heterogeneity and overlap between various repeat expansion disorders. Moreover, since both methods require separate primers/probes for each STR, parallel analysis of multiple candidates in a single assay is not possible.

Southern blot assays are regarded as the gold-standard for detecting large polynucleotide repeat expansions, but this method is time-consuming, inefficient, costly and requires large quantities (up to 10 μg) of high-quality DNA for a single analysis [4]. In certain STR expansions, Southern blotting has been replaced by RP-PCR, which is cheaper and more efficient [151]. However, because the highly repetitive region is amplified and then fragmented into shorter reads, PCR stutter errors make it difficult to accurately determine the length of an expanded repeat. Furthermore, in large repeats with high ‘GC’ content, repetitive flanking regions or flanking variants, it can be highly challenging to establish an effective diagnostic PCR assay. This is evident in testing regimes for *C9orf72*, which have not been standardised across labs [4]. Currently, optimised PCR methods can detect expanded repeat sizes up to 900 hexanucleotide repeats, However, accurate quantitative sizing may only be reported up to 140 repeats [26, 151].

Furthermore, while interruptions may be detected within a repeat, their exact motif may be challenging to determine [61]. Due to the high concentration of guanine-cystine (GC) content in some of these repeat and interruption motifs, there is a high chance of secondary structure formation and allelic dropout of PCR amplification leading to further sequencing errors [61, 75].

## Next generation sequencing

Next-generation sequencing (NGS) provides an alternative approach for genotyping STRs. STR expansions can be detected across the entire genome, using established short-read NGS platforms (e.g., Illumina), and a growing number of bioinformatics tools have been developed for this purpose (e.g., ExpansionHunter, LobSTR, RepeatSeq, HipSTR and GangSTR) [35, 57, 84, 112]. These tools also allow researchers to link STR regions in affected family members, making them good methods for identifying novel expansions, thereby leading to a recent wave of discoveries (as described earlier). The major advantage of whole-genome sequencing is that, in theory, all STRs in the genome are profiled simultaneously, as well as STR contraction and non-STR mutations, which may also be implicated in disease. While NGS remains relatively expensive, avoiding the need for repeated molecular testing on multiple targets means this can be cost effective, and will be increasingly competitive as sequencing prices continue to fall.

**Fig. 4:**
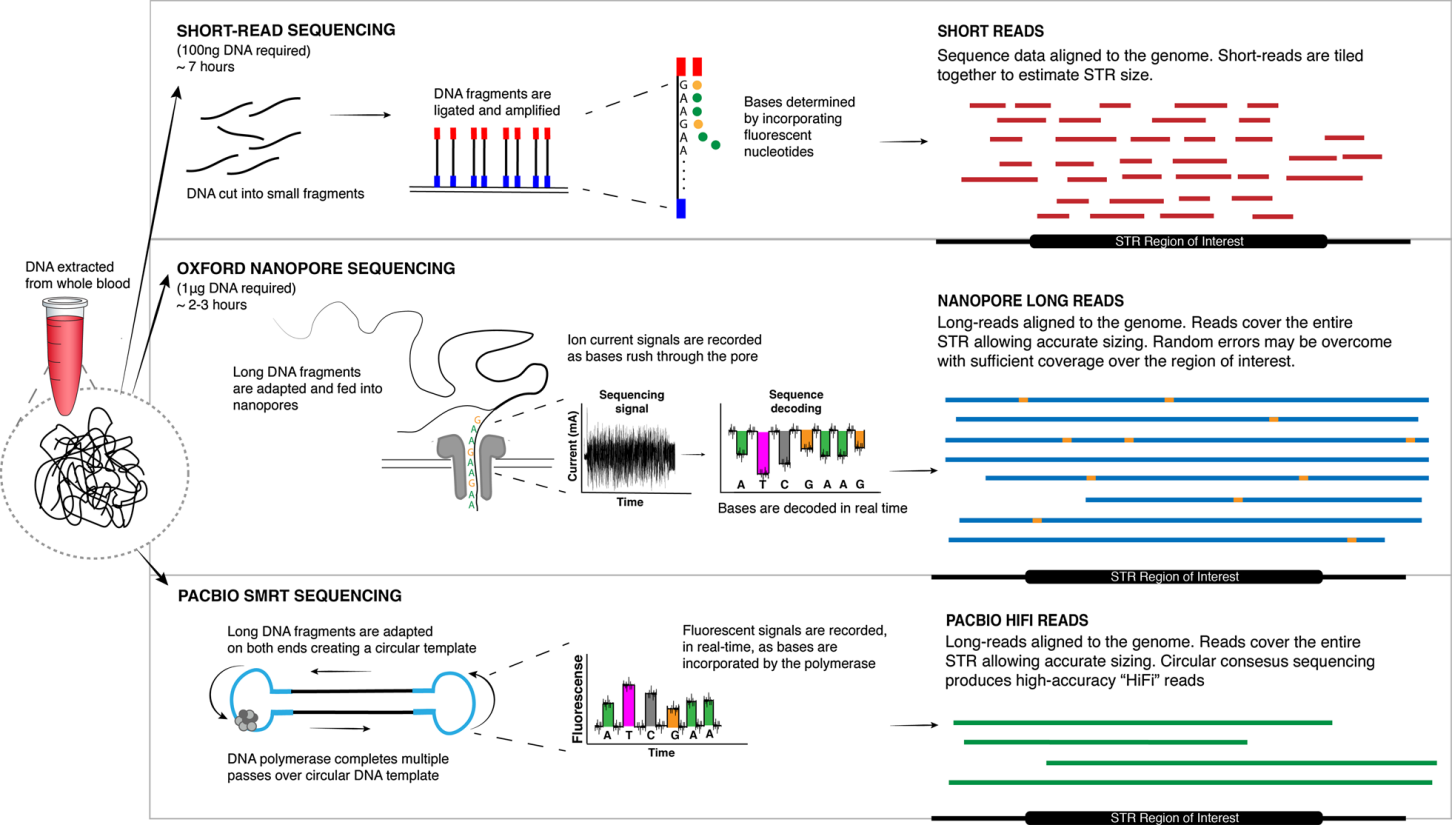
NGS and Long-read sequencing for diagnosing short tandem repeat expansions. Flow chart shows the use of short-read NGS and two long-read sequencing methods for genotyping STR expansions: PacBio single-molecule real-time (SMRT) sequencing and Oxford Nanopore Technology (ONT) long-read sequencing. The alignment of reads to the genome can be seen for all three methods; short-reads are ‘tiled’ together to estimate the repeat size and sequence, while long reads easily span repeat and flanking regions. Nanopore sequencing high error rates can be overcome via sufficient coverage

However, the utility of short-read NGS for repeat expansion diagnosis is hampered by several limitations. Firstly, highly repetitive and/or ‘GC’ rich genome regions are refractory to NGS library preparation, PCR amplification and sequencing, making it difficult to obtain sufficient coverage in many STR regions. PCR amplification during the library preparation can also introduce stutter errors, although this can be alleviated through the use of PCR-free library preparations [104]. Secondly, the repetitive nature of STR regions can cause ambiguous alignment or misalignment of short NGS reads to the reference genome. More fundamentally, the short-read length (~ 100–150 bp) of established NGS technologies is insufficient to span large STR expansions, making it impossible to precisely determine their length (see Fig. 4). Lastly, standard NGS does not detect epigenetic modifications, such as 5-methylcytosine, which are diagnostically important in some cases [132, 144]. Although NGS has proven useful for the discovery of new disease-related repeat expansions, these limitations have so far prevented widespread adoption of NGS for clinical diagnosis and replacement of low-throughout molecular tests like Southern blotting.

## Outlook: efficient and accurate diagnosis of repeat expansion disorders with long-read sequencing

For thorough evaluation of a suspected repeat expansion disorder, clinicians must be able to: (1) screen for all the relevant genes (including any newly discovered candidates); (2) accurately assess the size of any detected expansion and; (3) look for additional diagnostic or prognostic markers such as repeat interruptions and DNA methylation state. Emerging long-read sequencing platforms from Oxford Nanopore Technology (ONT) and Pacific Biosciences (PacBio) have the potential to address these requirements, while overcoming the limitations of conventional Illumina short-read sequencing platforms [84].

ONT devices measure the displacement of ionic current as a DNA strand passes through a biological nanopore and subsequently translate this data into DNA sequence information (see Fig. 4). ONT sequencing has no theoretical upper limit on read length, with > 10 kb average read length considered standard for genomic DNA sequencing and some examples achieving maximum read lengths in excess of 1 Mb [98]. Therefore, unlike for short-read NGS, individual ONT reads may span the entire length of large pathogenic repeat expansions (see Fig. 4 below). In one study, between 80 and 99.5% of reads successfully spanned expanded ‘GGCCTG’ repeats in *NOP56* (median 37 repeats) and ‘CCCCGG’ repeats in *C9orf72* (median 406 repeats), allowing direct measurement of STR lengths [36]. Nanopore reads currently exhibit relatively high sequencing error rates when compared to NGS, due to inaccuracies in the base-calling process, however, accurate consensus sequence determination is possible with sufficient coverage [70] and several studies have demonstrated accurate genotyping of repeat expansions with ONT [36, 46, 146]. Additionally, analysis of ONT signal data allows the methylation status of a given loci to be determined in parallel, providing an additional marker for the diagnosis of relevant repeat expansion disorders, such as FXS [46].

PacBio Single Molecule, Real-Time (SMRT) sequencing technology detects, in real-time, fluorescent signals from nucleotides as they are being incorporated to a single DNA template-polymerase [128]. SMRT sequencing achieves greater than 99% accuracy via circular consensus sequencing (CCS), whereby large DNA strands are ligated on either end to form a circular DNA molecule such that the DNA polymerase completes multiple passes of the same DNA fragment in a single read to achieve high coverage (average read-length 13.5 kb) [165]. An advantage of the long and highly accurate reads generated by PacBio SMRT sequencing, is the ability to resolve the STR length and sequence, as well as detecting and phasing possible variants in the surrounding regions. For example, a recent study developed a haplotype phasing protocol for the *HTT* gene using PacBio SMRT sequencing, enabling detection of relevant SNPs and ‘CAG’ expansions in *HTT* on the same amplicon [153]. Several new bioinformatics tools, such as IsoPhase [163], SHAPEIT4 [33] and NanoCaller [1], use long reads to accurately phase SNV, insertions and deletions. Thus, both ONT and PacBio SMRT technologies have the potential to replace current clinical molecular diagnostics by accurately generating reads spanning the length of large pathogenic repeat expansions.

Despite these promising recent developments, the computational analysis of long-read sequencing data to accurately genotype repeats is an active area of development, with several important hurdles yet to be overcome. Multiple software packages have been recently created for this purpose, including tandem-genotypes [106], NanoSatellite [31], STRique [46], RepeatHMM [93] and PacmonSTR [158], with each demonstrating the capability to measure the size of expanded STRs. However, discordant results between some tools [106] highlight the need for more rigorous benchmarking on a broad selection of different repeat types and sizes. Furthermore, the ability to resolve challenging cases such as STR interruptions, mixed conformations (e.g., the Māori-specific *RFC1* conformation [11]) and allelic differences in conformations, has yet to be demonstrated. Furthermore, the detection of novel pathogenic STR expansions remains another major unsolved challenge given the polymorphic nature of STRs and the vast STR diversity encountered in human populations [93, 106].

Whole-genome analysis with both ONT and PacBio long-read sequencing platforms is now feasible and will likely aid in the discovery of many novel disease-related STR expansions in the near future. For example, Sone and colleagues recently discovered a ‘GGC’ repeat in the *NOTCH2NLC* gene in 13 patients affected with NIID using long-read whole-genome sequencing combined with bioinformatics tool tandem-genotypes [146]. They then confirmed their findings with RP-PCR on positive and healthy controls. Similarly, a ‘TTTCA’ repeat expansion was discovered in *SAMD12* and linked to FAME1; the study used low-coverage (~ 10×) PacBio long-read sequencing with STR detection tools RepeatHMM and inScan to target the locus identified by linkage analysis [179]. It should also be noted that the ‘TTTCA’ expansion in the *SAMD12* gene was also discovered independently by Ishiura and colleagues, who used linkage analysis followed by repeat-primed PCR and Southern blotting to detect the expansion, then used PacBio to elucidate the motif structure [68].

Given the high cost and large data volumes generated using whole-genome, targeted sequencing of candidate genes represents a more viable and cost-effective pathway to clinical adoption. This requires the establishment of reliable methods for amplification-free enrichment and sequencing of long DNA fragments spanning STR regions.

One promising strategy involves the use of CRISPR-Cas9 guide-ribonucleoproteins (RNPs) for selective cleavage of target loci, followed by ligation of a magnetic adaptor that allows isolation of target molecules prior to PacBio SMRT sequencing [157]. To date, this method has been applied for genotyping STR expansions in *HTT*, *C9orf72*, *ATXN10* and *NOTCH2NLC* [146, 157]. ONT sequencing is amenable to an analogous strategy, where ONT sequencing adapters are directly ligated to Cas9 cleavage sites to enable their selective sequencing [46, 48]. In establishing this approach, Giesselmann et al. found a single ONT MinION flow-cell could generate greater than 40-fold coverage over the expanded ‘GGGGCC’ region in *C9orf72* [46], sufficient for accurate determination of repeat length. Furthermore, using their own raw signal algorithm termed STRique, they were able to profile ‘CpG’ methylation of the STR and its flanking regions, with hypermethylation observed at the *C9orf72* promoter in mutated alleles. In the study by Sone et al. mentioned above, they also used Cas9-mediated enrichment to achieve high sequencing depth (100–1795×) following their initial low-coverage whole-genome sequencing [146]. Furthermore, this method aided in identifying a ‘AAGGG’ repeat in a Japanese family in the *RFC1* gene as well as benign ‘TAAAA’ and ‘TAGAA’ expansions in *BEAN1* [114]. Cas9-mediated target enrichment is amenable to multiplexing, making it feasible to target multiple disease alleles in parallel, for more efficient and cost-effective diagnosis. For example, Tsai et al. demonstrated parallel enrichment of *C9orf72, HTT*, *FMR1* and *ATXN10,* achieving 150–2000-fold coverage depth with SMRT sequencing on all targets in a single assay [157]. This capability is advantageous from a diagnostic perspective, avoiding the need to order multiple tests, as is the case with standard molecular diagnostics.

Another recent innovation in ONT sequencing is programmable target selection, using ONT’s Read Until API. Via real-time identification and rejection of off-target DNA fragments, Read Until affords enriched sequencing depth across target regions of the user’s choice without requiring any upstream molecular target enrichment [80, 124]. One unpublished study has already applied this new approach to the detection of repeat expansions, simultaneously determining repeat size and methylation status in patients with pathogenic expansions in *FMR1*, *FXN*, *ATXN3*, *ATXN8*, or *XYLT1* [105]. Besides the obvious advantage in avoiding cumbersome molecular methods of target enrichment, the Read Until method allows hundreds or even thousands of candidate loci to be targeted in parallel, and the specific set of targets can be easily customised for a given patient depending on their phenotype and family history. These advantages could see programmable ONT sequencing become the preferred method for both diagnosis and discovery of repeat expansion disorders in the near future.

## Conclusions

Short tandem repeat expansion disorders are highly important in human disease, particularly in the field of neurology. The list of repeat expansion disorders is currently over 40 and growing rapidly. This is highlighted by the recent findings that several important disorders in neurology (such as CANVAS and NIID) have been found to be caused by short tandem repeat expansions. The established methods for diagnosing these disorders are cumbersome and time consuming. However, long-read sequencing offers the opportunity to transform the detection of repeat expansion disorders, allowing for rapid and accurate genotyping. This would provide a more in-depth understanding of healthy and pathogenic repeat ranges, transmission and clinical anticipation, and the role of interruptions. Further research is required to overcome the technical hurdles and fully exploit the potential of long-read sequencing. Additionally, cost-effectiveness studies are required to compare the cost associated with long-read sequencing approaches to traditional methods of detecting repeat expansion disorders prior to widespread use in clinical practice.

## Data Availability

Data sharing is not applicable to this article as no datasets were generated or analysed during the current study.
